# Supplementary material for: Myosin IIA and formin dependent mechanosensitivity of filopodia adhesion
Source: Nat Commun. 2019 Aug 9;10:3593. doi: 10.1038/s41467-019-10964-w (PMC6689027; doi:10.1038/s41467-019-10964-w)
Supplement: Supplementary file 1 — Supplementary Information [file 41467_2019_10964_MOESM1_ESM.pdf]

Supplementary Information for

Myosin IIA and formin dependent mechanosensitivity of filopodia adhesion

N.O. Alieva et al.

This PDF file includes:

Supplementary text 1: Mechanical behavior and adhesion of filopodia induced by active Cdc42 and formin mDia2

Supplementary Note 2: Estimation of forces developed by individual myosin IIA filament

Supplementary Note 3: Expression vectors used in this study

Supplementary Figures 1 to 7 with the legends

Supplementary References

Other Supplementary Information for this manuscript includes the following:

Supplementary Movies 1 to 27

Legends to Supplementary Movies 1 to 27

Supplementary note 1. Mechanical behavior and adhesion of filopodia induced by active Cdc42 and formin mDia2

It should be noted that all experiments described above were performed on filopodia induced by overexpressing myosin X in HeLa-JW or Cos-7 cells. Therefore, strictly speaking, our conclusions are only valid for myosin X induced filopodia. Our choice of experimental system is justified by the fact that myosin X has been shown to be a universal component of filopodia [1] and it is often overexpressed in metastasizing cancer cells[2, 3]. Nevertheless, to explore the generality of our conclusions, we performed several pilot experiments with filopodia induced in the same cell types by activation of other components. In particular, we investigated filopodia induced by constitutively active small G-protein Cdc42 (Cdc42 Q61L), an upstream regulator of filopodia formation[4, 5], and constitutively active formin mDia2 (mDia2 ΔDAD), an activator of actin polymerization localized to filopodia tips.

In Cos-7 cells expression of either mDia2 ΔDAD-GFP or GFP-Cdc42 Q61L resulted in formation of long and often wavy filopodia (Supplementary Fig. 7a and b). The dynamics of these filopodia differed from those of filopodia induced by overexpression of myosin X; the lifetimes of both mDia2 ΔDAD- and Cdc42 Q61L-induced filopodia were an order of magnitude longer than filopodia induced by myosin X (Supplementary Fig. 7c and d). Remarkably, co-expression of myosin IIA with mDia2 ΔDAD formin significantly increased the lifetime of mDia2 ΔDAD-induced filopodia, while co-expression of myosin IIA with Cdc42 Q61L did not affect the average lifetime of Cdc42 Q61L-induced filopodia. This suggests that filopodia induced by active formin are myosin IIA dependent, qualitatively similar to filopodia

induced by myosin X, while filopodia induced by active Cdc42 demonstrate different behavior.

In HeLa-JW cells, Cdc42-induced filopodia attached to laser-trapped fibronectin-coated beads also demonstrated qualitatively different behavior as compared to filopodia induced by overexpression of myosin X. The Cdc42-induced filopodia did not show sustained growth upon pulling force application and withdrew the beads out of the trap soon after the start of stage movement (Supplementary Movie 26). The response to pulling force of filopodia induced in HeLa-JW cells by active formin mDia2 was also different from that of filopodia induced by myosin X. These filopodia essentially grew independently of pulling and often continued to grow further bypassing the bead location (Supplementary Movie 27). Altogether these preliminary data show that filopodia formed upon stimulation by different inducers can demonstrate different dependence on pulling forces and myosin IIA than filopodia induced by myosin X. More in-depth studies of these types of filopodia are beyond the scope of present work.

Supplementary Note 2. Estimation of forces developed by individual myosin IIA filament

The force generated by one bipolar myosin IIA filament (consisting of about 30 individual myosin molecules, n = 15 at each side [6]) can be estimated based on the stall force for an individual myosin IIA molecule (f~ 3.4 pN [7]) and duty ratio (d = 5-11% [8]) as 2.6-5.6 pN. Indeed, at each time instant there will be on average ~ $n \times d$ = 0.8-1.7 active myosin IIA heads on each side of the bipolar filament, generating a pulling force on surrounding actin filaments. Taking into account that each myosin IIA

molecule produces ~ f = 3.4 pN force, the total force, F, created by a single bipolar myosin IIA filament will be: F = f×n×d = 2.6-5.6 pN. This value is consistent with pulling forces generated by individual filopodia as measured in our experiments.

Supplementary Note 3. Expression vectors encoding fluorescent fusion proteins used in this study

GFP-myosin X and GFP-Myo10 ΔFERM [9, 10] (gift from Dr. R. Cheney, University of North Carolina Medical School, USA), reduced size myosin X construct (without 3'UTR) derived from GFP-myosin X was sub-cloned into mApple-C1 cloning vector backbone (M. Davidson collection, Florida State University, USA, kindly provided by Dr. P. Kanchanawong, MBI, NUS, Singapore) using KpnI and ApaI restriction sites, F-tractin-tdTomato [11] (gift from Dr. M. J. Schell, Uniformed Services University, Bethesda, Maryland, USA), mCherry-Utrophin [12, 13] kindly provided by Dr. W. Bement (University of Wisconsin, Madison, USA), mTagBFP-Lifeact (M. Davidson collection, Florida State University, USA (Addgene)), PAmCherry-β-actin was kindly provided by Dr. V. Verkhusha, Albert Einstein College of Medicine, New York, NY, USA). myosin II regulatory light chain (RLC)-GFP [14] (gift from Drs. W. Wolf and R. Chisholm, Northwestern University, Chicago, Illinois, USA), myosin IIA-GFP heavy chain (HC) was sub-cloned from pTRE-GFP-NMHCIIA (from Dr. R. Adelstein, NHLBI, NIH, USA) into pEGFP-C3 by MluI and HindIII cloning sites (cloned by Dr. M. Tamada, M. Sheetz laboratory), GFP-myosin IIA N93K [15] was kindly provided by Dr. Vicente-Manzanares (Universidad Autónoma de Madrid, Madrin, Spain), Emerald-myosin IIB, mCherry-myosin IIA HC, mCherry-VASP and mCherry-talin (M. Davidson collection, Florida State University, kindly provided by Dr. P. Kanchanawong, MBI, NUS, Singapore), full-length mDia2 was sub-cloned (by Dr. X.

Shao, A. Bershadsky laboratory) from GFP-C1-mDia2 [16] (gift from Dr. S. Narumiya, Faculty of Medicine, Kyoto University, Japan) into mCherry-C1 vector (Clontech). To obtain active mDia2 ΔDAD-GFP clone, 1-1036 amino acids region of mDia2 was cloned by PCR amplification using GFP-C1-mDia2 as a template, and subsequently cloned into GFP-N1 plasmid (Clontech), GFP-Cdc42 Q61L kindly provided by Dr. J. Brugge (Harvard University, Boston, MA, USA). All cell culture materials were obtained from Life Technologies.

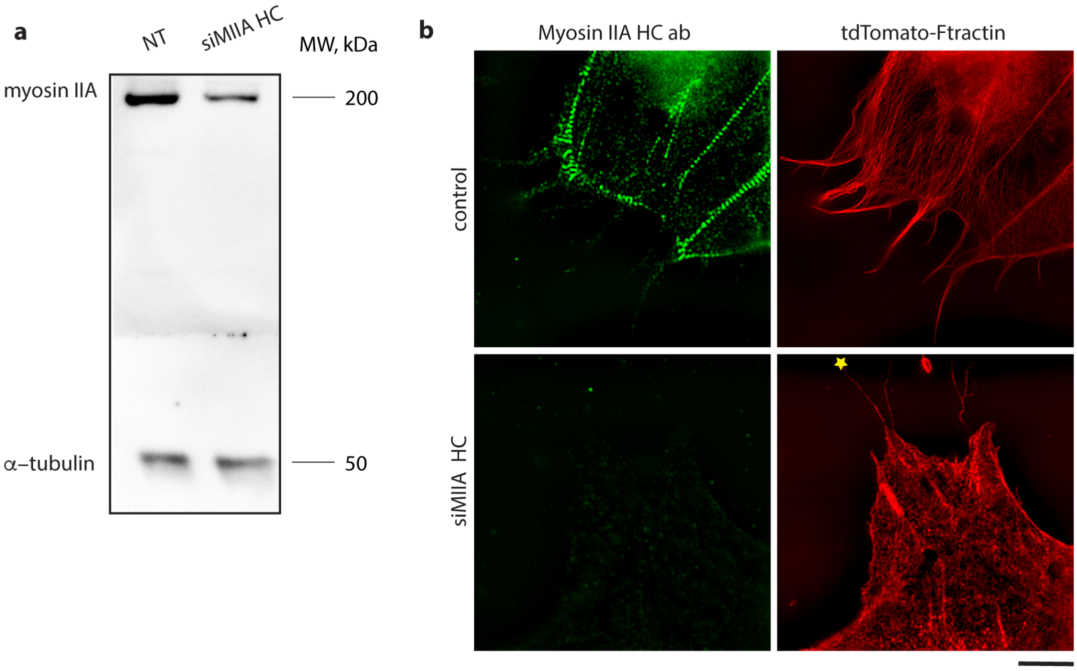

Supplementary Figure 1

siRNA knockdown of myosinIIA

(a) Immunoblots of myosin IIA in non-targeted (NT) and knockdown (siMIIA HC) HeLa-JW cells. α-tubulin was used as loading control. **(b)** siMIIA HC knockdown cells did not contain either myosin IIA (left bottom) or prominent actin stress fibers (right bottom), but still contained filopodia. The filopodium attached to the bead, which was stretched in one of our optical trap experiments, is indicated by an asterisk. Images were obtained with SDCM. Scale bar, 10 μm.

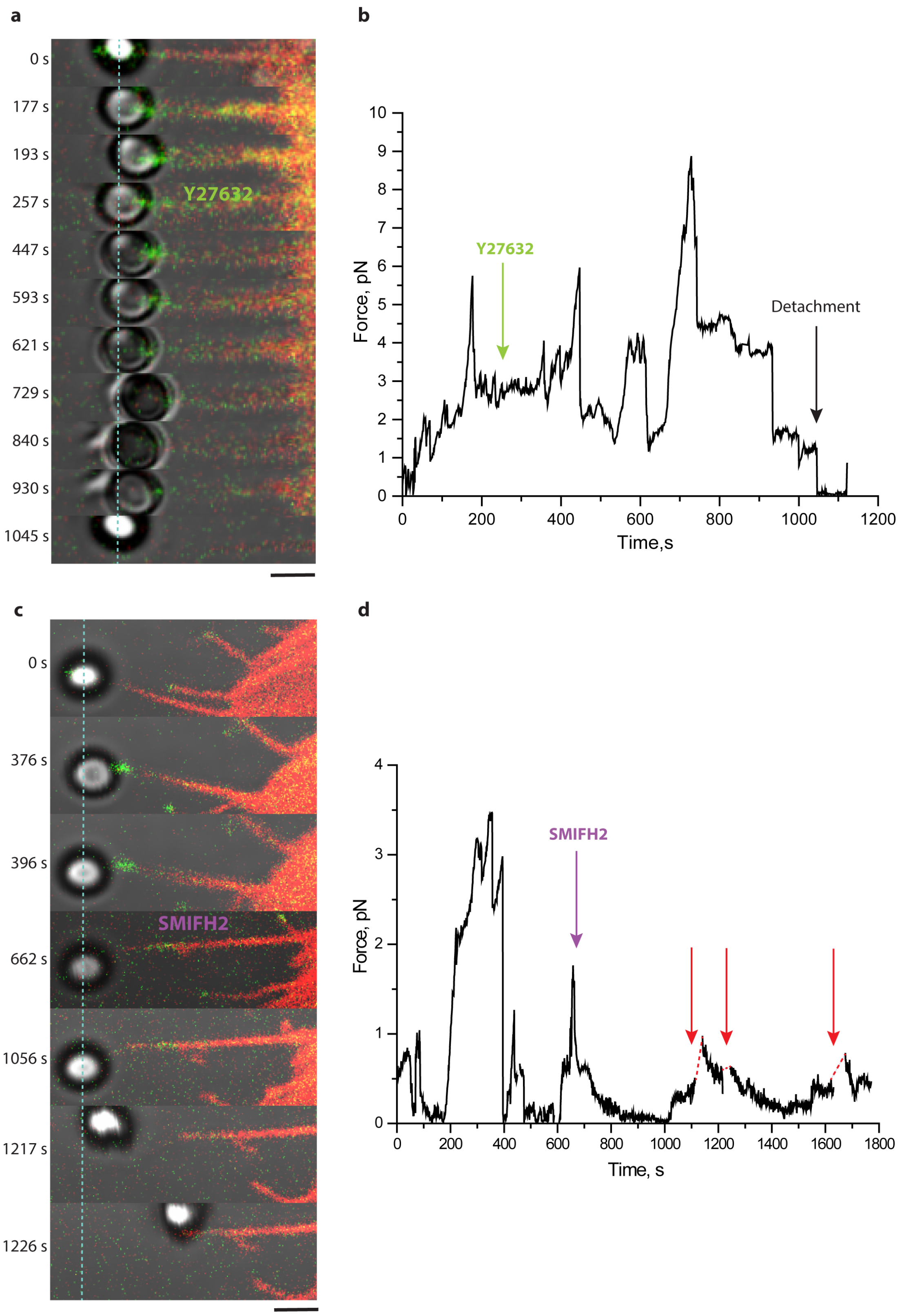

Supplementary Figure 2

Immediate effects of ROCK inhibitor, Y-27632, and formin inhibitor, SMIFH2, on force-induced filopodia growth and adhesion

Fibronectin-coated beads trapped by laser tweezers were attached to the filopodia tips of HeLa-JW cells. Filopodia growth was then induced through the generation of pulling force, which resulted from the movement of the microscope stage, as shown in Fig. 3. 30 μM of Y-27632 **(a-b)** or 40 μM of SMIFH2 **(c-d)** were added at about 4 and 11 min following the start of the stage movement, respectively. The positions of the trapped beads during the filopodia growth are shown in (a) and (c). The intensity of actin labeling in (a) was relatively low and the apparent "disappearance" of actin in the late frames was a result of photobleaching. Scale bars, 2 μm. See Supplementary Movies 15 and 22. (b) Dynamics of the forces exerted by the filopodia on the beads after addition of Y-27632 (green arrow). The transient increase of the force up to 10 pN around 730 s was followed by a gradual drop to zero at 932-1045 s. Detachment of the bead indicated by black arrow. Note that this experiment differs from the experiments summarized in Fig. 4f, in which the cells were pretreated by different inhibitors for more than 10 min. The development of a high force of 10 pN detected in this experiment may reflect a transient force imbalance during the immediate cell reaction to drug application. (d) Dynamics of the forces exerted by the filopodia on the beads after addition of SMIFH2 (purple arrow). Moments when the trap was switched off are indicated by red arrows. In this experiment, the bead remained associated with the filopodium via the membrane tether (see Supplementary Movie 22).

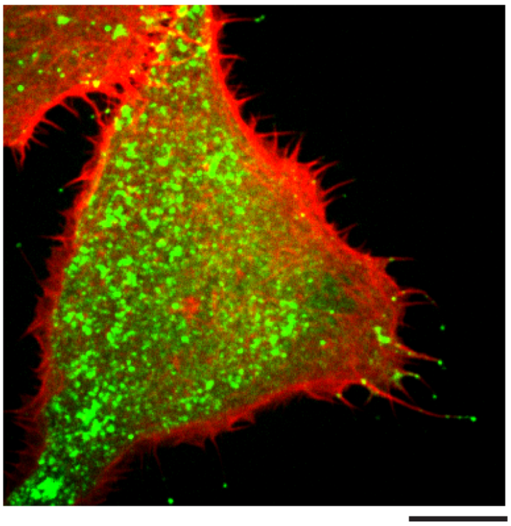

Supplementary Figure 3

Effect of formin inhibitor SMIFH2 on myosin X induced filopodia

Image of HeLa-JW cell, labeled with GFP-myosin X and mCherry-Utrophin, that was treated with 20 μM SMIFH2 formin inhibitor for 2 hours**.** Note that the majority of the filopodia do not contain myosin X. The image was obtained using SDCM. Scale bar, 10 μm. Scoring of filopodia in 14 cells taken in 2 independent experiments revealed that only 25% of filopodia contained myosin X at their tips.

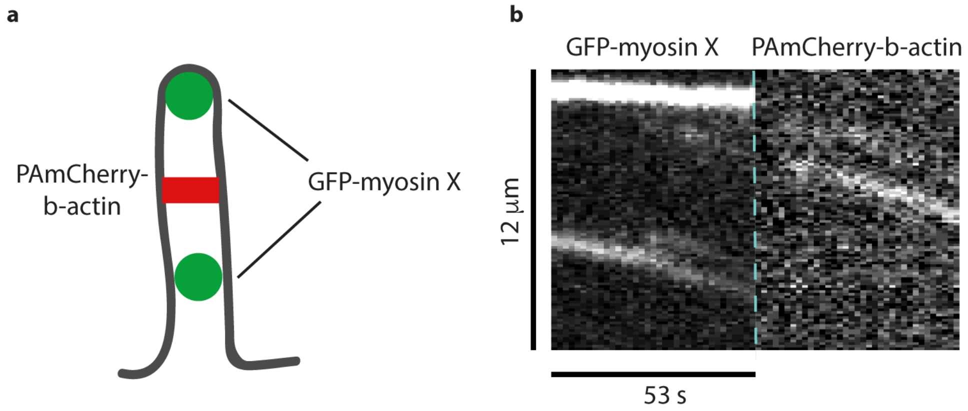

Supplementary Figure 4

Retrograde movement of myosin X-positive patches coordinates with movement of photoactivated β-actin inside filopodia

(a) Schematic depiction of myosin X-induced filopodia in photoactivation experiments. Green circles represent myosin X-positive patches while the red rectangle indicates the area of photoactivation. **(b)** Kymograph analysis of the retrograde movement of myosin X and photoactivated actin in the same filopodia. Left panel corresponds to GFP-myosin X and shows immobile tip of the filopodium (upper line) and retrograde movement of myosin X (tilted line). Right panel represents the retrograde movement of photoactivated PAGFP-β-actin (tilted line) in the same filopodia. Note that the rate of retrograde movements of myosin X and actin are similar and were equal to 37 nm/s. See also Supplementary Movie 19.

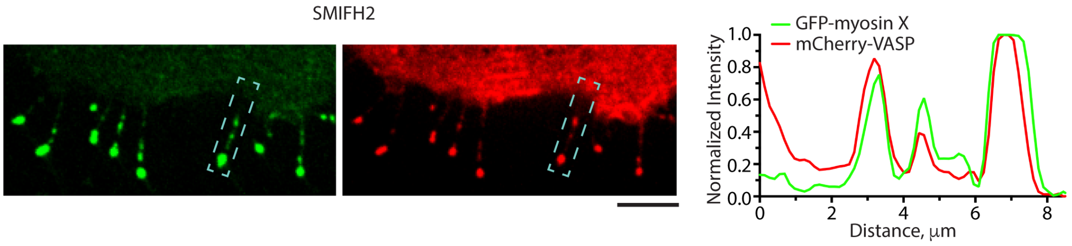

Supplementary Figure 5

Localization of VASP at myosin X patches in cells treated with formin inhibitor

Distribution of GFP-myosin X (green, left panel) and mCherry-VASP (red, central panel) in the same HeLa-JW cell 1.5 hours after addition of 20 μM SMIFH2. Right panel: the line scans through the boxed area, showing the co-distribution of myosin X and VASP. Fluorescent intensities are normalized to their maximal values. Scale bar, 5 μm. Images were obtained with SDCM.

a b

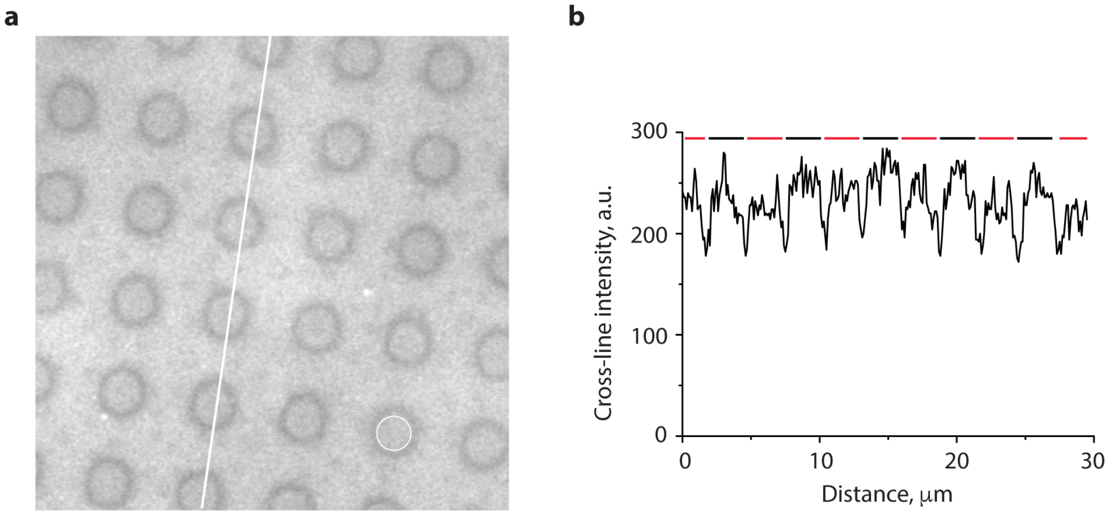

Supplementary Figure 6

(a) A fluorescence image of a hybrid substrate containing a RGD ligand which is tethered to PLL-PEG polymer (background) and a supported lipid bilayer (SLB) surface (circles) via a biotin- Dylight-405 NeutrAvidin conjugation. **(b)** RGD intensity profile along the white line shown in (a). The red and black lines at the top of the curve mark the SLB and PLL-PEG regions, respectively, showing a similar concentration of RGD ligands in both regions.

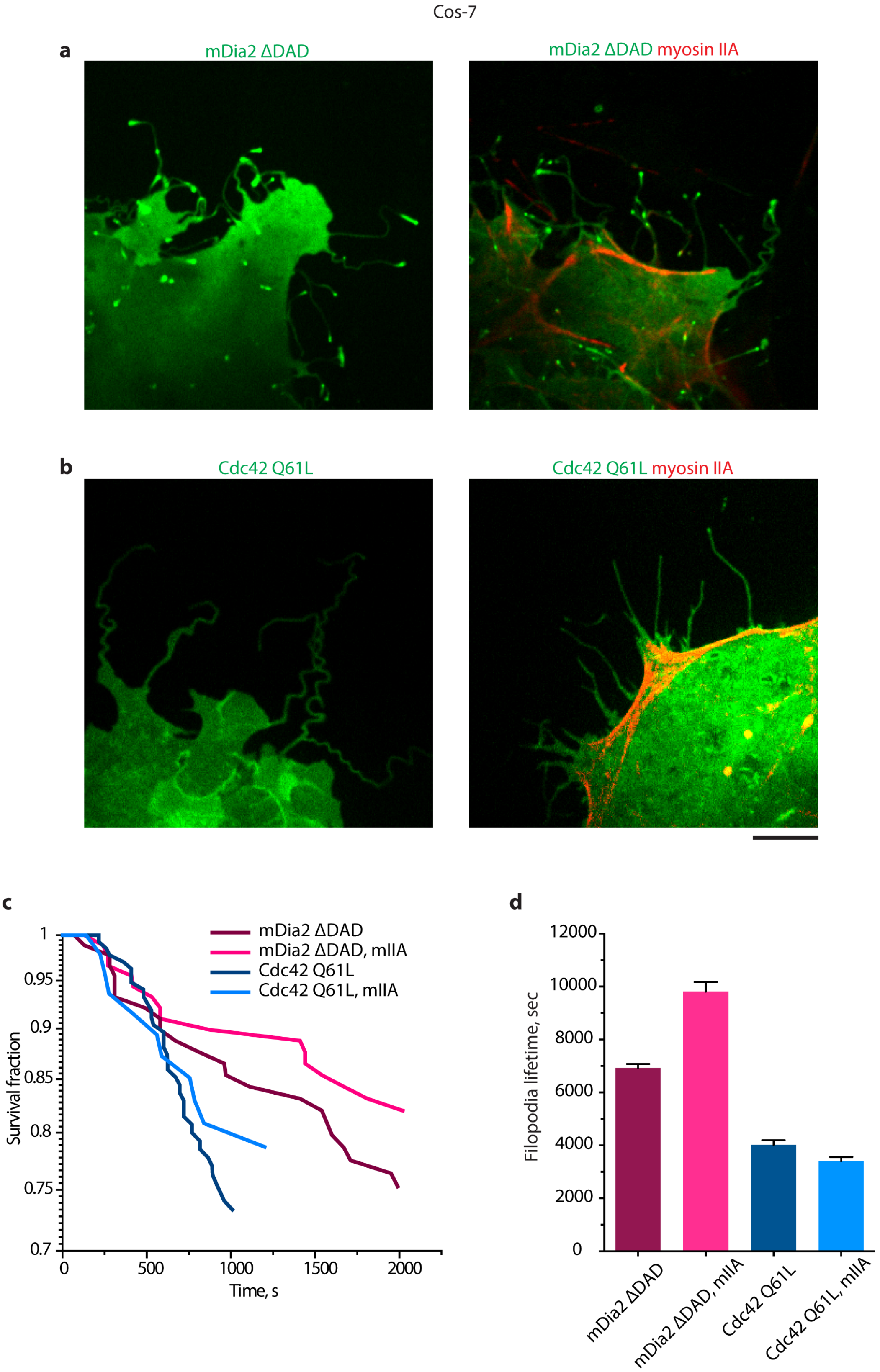

Supplementary Figure 7

Myosin IIA dependence of survival time of filopodia induced by constitutively active mDia2 or constitutively active Cdc42

(a-b) Cos-7 cells expressing mDia2 ΔDAD-GFP (a, left panel), GFP-Cdc42 Q61L (b, left panel), GFP-mDia2 ΔDAD and Cherry-myosin IIA HC (a, right panel) and GFP-Cdc42 Q61L and Cherry-myosin IIA HC (b, right panel). Images were obtained on SDCM. Scale bar, 10 µm. **(c)** Dynamics of survival fractions of the filopodia cohorts in Cos-7 cells transfected with the indicated constructs. **(d)** Lifetimes of filopodia calculated from the graphs shown in (c) by fitting them to exponential decay function. Calculated lifetime values (s) indicated in the graph (from left to right) were (mean ± s.d.): 6927.8 ± 144.9 (n = 89, 5 cells), 9808.7 ± 360.0 (n = 89, 4 cells), 4018.2 ± 177.2 (n = 89, 4 cells) and 4618.9 ± 199.9 (n = 47, 12 cells).

Supplementary references

1. Kerber, M.L. & Cheney, R.E. Myosin-X: a MyTH-FERM myosin at the tips of filopodia. *J Cell Sci* **124**, 3733-3741 (2011).
2. Cao, R. *et al.* Elevated expression of myosin X in tumours contributes to breast cancer aggressiveness and metastasis. *Br J Cancer* **111**, 539-550 (2014).
3. Arjonen, A. *et al.* Mutant p53-associated myosin-X upregulation promotes breast cancer invasion and metastasis. *J Clin Invest* **124**, 1069-1082 (2014).
4. Nobes, C.D. & Hall, A. Rho, rac, and cdc42 GTPases regulate the assembly of multimolecular focal complexes associated with actin stress fibers, lamellipodia, and filopodia. *Cell* **81**, 53-62 (1995).
5. Kozma, R., Ahmed, S., Best, A. & Lim, L. The Ras-related protein Cdc42Hs and bradykinin promote formation of peripheral actin microspikes and filopodia in Swiss 3T3 fibroblasts. *Mol Cell Biol* **15**, 1942-1952 (1995).
6. Billington, N., Wang, A., Mao, J., Adelstein, R.S. & Sellers, J.R. Characterization of three full-length human nonmuscle myosin II paralogs. *J Biol Chem* **288**, 33398-33410 (2013).
7. Hundt, N., Steffen, W., Pathan-Chhatbar, S., Taft, M.H. & Manstein, D.J. Load-dependent modulation of non-muscle myosin-2A function by tropomyosin 4.2. *Sci Rep* **6**, 20554 (2016).
8. Kovacs, M., Wang, F., Hu, A., Zhang, Y. & Sellers, J.R. Functional divergence of human cytoplasmic myosin II: kinetic characterization of the non-muscle IIA isoform. *J Biol Chem* **278**, 38132-38140 (2003).
9. Berg, J.S. & Cheney, R.E. Myosin-X is an unconventional myosin that undergoes intrafilopodial motility. *Nat Cell Biol* **4**, 246-250 (2002).
10. Bohil, A.B., Robertson, B.W. & Cheney, R.E. Myosin-X is a molecular motor that functions in filopodia formation. *Proc Natl Acad Sci U S A* **103**, 12411-12416 (2006).
11. Schell, M.J., Erneux, C. & Irvine, R.F. Inositol 1,4,5-trisphosphate 3-kinase A associates with F-actin and dendritic spines via its N terminus. *J Biol Chem* **276**, 37537-37546 (2001).
12. Lin, A.Y., Prochniewicz, E., James, Z.M., Svensson, B. & Thomas, D.D. Large-scale opening of utrophin's tandem calponin homology (CH) domains upon actin binding by an induced-fit mechanism. *Proc Natl Acad Sci U S A* **108**, 12729-12733 (2011).

13. Winder, S.J. *et al.* Calmodulin regulation of utrophin actin binding. *Biochem Soc Trans* **23**, 397S (1995).
14. Kengyel, A., Wolf, W.A., Chisholm, R.L. & Sellers, J.R. Nonmuscle myosin IIA with a GFP fused to the N-terminus of the regulatory light chain is regulated normally. *J Muscle Res Cell Motil* **31**, 163-170 (2010).
15. Vicente-Manzanares, M., Ma, X., Adelstein, R.S. & Horwitz, A.R. Non-muscle myosin II takes centre stage in cell adhesion and migration. *Nat Rev Mol Cell Biol* **10**, 778-790 (2009).
16. Watanabe, S. *et al.* mDia2 induces the actin scaffold for the contractile ring and stabilizes its position during cytokinesis in NIH 3T3 cells. *Mol Biol Cell* **19**, 2328-2338 (2008).
