# Supplementary material for: Myosin IIA and formin dependent mechanosensitivity of filopodia adhesion
Source: Nat Commun. 2019 Aug 9;10:3593. doi: 10.1038/s41467-019-10964-w (PMC6689027; doi:10.1038/s41467-019-10964-w)
Supplement: Supplementary file 3 — Reporting Summary [file 41467_2019_10964_MOESM3_ESM.pdf]

nature research

Corresponding author(s): A. Bershadsky

Last updated by author(s): Jan 17, 2019

Reporting Summary

Nature Research wishes to improve the reproducibility of the work that we publish. This form provides structure for consistency and transparency in reporting. For further information on Nature Research policies, see Authors & Referees and the Editorial Policy Checklist.

Statistics

For all statistical analyses, confirm that the following items are present in the figure legend, table legend, main text, or Methods section.

n/a	Confirmed	
☐	☒	The exact sample size (*n*) for each experimental group/condition, given as a discrete number and unit of measurement
☐	☒	A statement on whether measurements were taken from distinct samples or whether the same sample was measured repeatedly
☐	☒	The statistical test(s) used AND whether they are one- or two-sided<br>*Only common tests should be described solely by name; describe more complex techniques in the Methods section.*
☐	☒	A description of all covariates tested
☐	☒	A description of any assumptions or corrections, such as tests of normality and adjustment for multiple comparisons
☐	☒	A full description of the statistical parameters including central tendency (e.g. means) or other basic estimates (e.g. regression coefficient) AND variation (e.g. standard deviation) or associated estimates of uncertainty (e.g. confidence intervals)
☐	☒	For null hypothesis testing, the test statistic (e.g. *F*, *t*, *r*) with confidence intervals, effect sizes, degrees of freedom and *P* value noted<br>*Give P values as exact values whenever suitable.*
☒	☐	For Bayesian analysis, information on the choice of priors and Markov chain Monte Carlo settings
☒	☐	For hierarchical and complex designs, identification of the appropriate level for tests and full reporting of outcomes
☒	☐	Estimates of effect sizes (e.g. Cohen's *d*, Pearson's *r*), indicating how they were calculated

Our web collection on statistics for biologists contains articles on many of the points above.

Software and code

Policy information about availability of computer code

Data collection	Software provided by Nikon (NIS-Elements), Velocity, MetaMorph for images / video acquisition
Data analysis	Data analysis was done by using Origin, GraphPad Prism, MetaMorph, Python, MatLab, Image J, and Imaris software packages

For manuscripts utilizing custom algorithms or software that are central to the research but not yet described in published literature, software must be made available to editors/reviewers. We strongly encourage code deposition in a community repository (e.g. GitHub). See the Nature Research guidelines for submitting code & software for further information.

Data

Policy information about availability of data

All manuscripts must include a data availability statement. This statement should provide the following information, where applicable:

- Accession codes, unique identifiers, or web links for publicly available datasets
- A list of figures that have associated raw data
- A description of any restrictions on data availability

The authors declare that all data supporting the findings of this study are available within the article and its Supplementary Information or from the corresponding author upon reasonable request.

Field-specific reporting

Please select the one below that is the best fit for your research. If you are not sure, read the appropriate sections before making your selection.

☒ Life sciences ☐ Behavioural & social sciences ☐ Ecological, evolutionary & environmental sciences

October 2018

Life sciences study design

All studies must disclose on these points even when the disclosure is negative.

Sample size	No sample-size calculations were performed. Sample size was determined to be adequate based on the magnitude and consistency of measurable differences between groups.
Data exclusions	Data were only excluded for failed experiments, reasons for which included failure to complete experiment with optical trap due to accidental attachment of optically trapped bead to glass bottom surface of cell culture chamber.
Replication	Replicate experiments were successful
Randomization	Not applicable. Data for analysis were collected either on same cell before and after application of experimental conditions (mechanical pulling or treatment with drugs) and all the outcomes of such experiments (except failures) were recorded or on parallel sets of experimental cells (control and treatments), which were randomly selected for followed analysis. Majority of experiments of the same type were conducted in two or more different days.
Blinding	Investigators were not blinded during experiments.

Reporting for specific materials, systems and methods

We require information from authors about some types of materials, experimental systems and methods used in many studies. Here, indicate whether each material, system or method listed is relevant to your study. If you are not sure if a list item applies to your research, read the appropriate section before selecting a response.

Materials & experimental systems

n/a	Involved in the study	
☐	☒	Antibodies
☐	☒	Eukaryotic cell lines
☒	☐	Palaeontology
☒	☐	Animals and other organisms
☒	☐	Human research participants
☒	☐	Clinical data

Methods

n/a	Involved in the study	
☒	☐	ChIP-seq
☒	☐	Flow cytometry
☒	☐	MRI-based neuroimaging

Antibodies

Antibodies used	1. Primary rabbit antibodies to the myosin IIA tail domain (M8064, Sigma-Aldrich), 2. Anti-α-tubulin, mouse monoclonal antibody (T6199, Sigma-Aldrich)
Validation	Validation on applications in which these antibodies have been used successfully, and the associated peer-reviewed papers, are provided on Sigma-Aldrich web pages: https://www.sigmaaldrich.com/catalog/product/sigma/m8064?lang=en&region=SG https://www.sigmaaldrich.com/catalog/product/sigma/t6199?lang=en&region=SG

Eukaryotic cell lines

Policy information about cell lines

Cell line source(s)	Cos-7 cells line was obtained from ATCC (ATCC, CRL-1651). Hela-JW - was obtained from the laboratory of B. Geiger, Weizmann Institute of Science, Israel.
Authentication	Cos-7 cells were authenticated by ATCC. HeLa-JW cells were not re-authenticated by authors. Authors declare that after the original frozen cell vials were obtained from the sources, they were rapidly (no more than after 1-2 passages) propagated into in-house-made frozen aliquots. Both cell lines undergo no more than 25 passages before new aliquot thaws to be used in experiments.
Mycoplasma contamination	Both cells lines, which were used in this study, were tested negative in mycoplasma contamination test.
Commonly misidentified lines (See ICLAC register)	HeLa-JW and Cos-7 are not found in ICLAC data base.
