# Supplementary material for: Myosin IIA and formin dependent mechanosensitivity of filopodia adhesion
Source: Nat Commun. 2019 Aug 9;10:3593. doi: 10.1038/s41467-019-10964-w (PMC6689027; doi:10.1038/s41467-019-10964-w)
Supplement: Supplementary file 4 — Description of Additional Supplementary Files [file 41467_2019_10964_MOESM4_ESM.pdf]

Description of Additional Supplementary Files

Supplementary Movie 1

Myosin IIA filaments at the bases of filopodia of HeLa-JW cell

This movie corresponds to the Fig. 1c. HeLa-JW ce1ls were transfected with mApple-myosin X (red), myosin II RLC-GFP (green), and mTagBFP-Lifeact (blue). The duration of the movie was 15 min 50 s. It was recorded at 0.003 fps and displayed at 7 fps.

Supplementary Movie 2

Cos-7 cell expressing GFP-myosin X (shown in red)

This movie corresponds to the Fig. 1f. The duration of the movie was 4 min 8 s. It was recorded at 0.5 fps and displayed at 18 fps.

Supplementary Movie 3

Myosin IIA filaments in Cos-7 cell, cell 1

This movie corresponds to the Fig. 1g. Cos-7 cell were expressing mApple-myosin X (red) and Myosin IIA-GFP (green). The duration of the movie was 4 min 5 s. It was recorded at 0.2 fps and displayed at 7.5 fps.

Supplementary Movie 4

Mutant myosin IIA in Cos-7 cell

This movie corresponds to the Fig. 1h. Cos-7 cell were expressing mApple-myosin X (red) and GFP-Myosin IIA N93K (green). The duration of the movie was 4 min 5 s. It was recorded at 0.2 fps and displayed at 7.5 fps.

Supplementary Movie 5

Myosin IIB in Cos-7 cell

This movie corresponds to the Fig. 1i. Cos-7 cell were expressing mApple-myosin X (red) and Emerald-myosin IIB (green). The duration of the movie was 3 min 15 s. It was recorded at 0.2 fps and displayed at 7.5 fps.

Supplementary Movie 6

Myosin IIA filaments in Cos-7 cell, cell2

This movie corresponds to the Fig. 2a. Cos-7 cell were expressing mApple-myosin (red) X, Myosin IIA-GFP (green) and mTagBFP-Lifeact (blue). This cell was used for data analysis for local effect of myosin IIA at the bases of filopodia (marked with one dot on graph on Fig. 2d). The duration of the movie was 4 min 1 s. It was recorded at 0.6 fps and displayed at 18 fps.

Supplementary Movie 7

Myosin X ΔFERM in filopodia of Cos-7 cell

Cos-7 cell were transfected with GFP-myosin X ΔFERM (shown in red). The duration of the movie was 4 min 5 s. It was recorded at 0.2 and displayed 7.5 fps. Movie was obtained by SIM.

Supplementary Movie 8

Myosin IIA and myosin X ΔFERM in filopodia of Cos-7 cell

Cos-7 cell were transfected with Myosin IIA-GFP (green) and GFP-myosin X ΔFERM (red). The duration of the movie was 4 min 5 s. It was recorded at 0.2 fps and displayed at 7.5 fps. Movie was obtained by SIM.

Supplementary Movie 9

3D view of the fixed myosin X-induced filopodium attached to a 2 μm fibronectin-coated bead

HeLa-JW cell was transfected with GFP-myosin X and F-Tractin-tdTomato. Single filopodium was attached to optically trapped bead. After pulling of the filopodium with the optical trap for several minutes, the cell was fixed by addition of 20% PFA (Tousimis) directly to the chamber up to final concentration of 2%. Image and position of the bead were reconstructed from bright field image. Note the clearly visible integrity of the actin core (red). See Methods for details on experiments using optical trap.

Supplementary Movie 10

Dynamics of force-induced growth of GFP-myosin X-induced filopodia of HeLa-JW cell, control filopodia 1

Sustained filopodial growth induced by pulling force generated by the optically trapped microbead coated with fibronectin. The frames from this movie, and the kymograph based on it, are shown in Fig. 3b and c respectively. The duration of the entire movie is 26 min 52 s. It was recorded at 0.5 fps and displayed at 25 fps.

Supplementary Movie 11

Dynamics of force-induced growth of GFP-myosin X-induced filopodia, control filopodia 2

The experiment was analogous to that described in the legend to Fig. 3a. This HeLa-JW cell was used as a control for experiments with inhibitors presented in Fig. 4a.

The duration of the movie is 17 min 8 s. It was recorded at 0.5 fps with a display rate of 15 fps.

Supplementary Movie 12

Pulling of GFP-myosin X-induced filopodia using concanavalin A-coated beads

Pulling force was applied to a filopodium of HeLa-JW cell via a concanavalin-A-coated laser-trapped bead by moving the microscope stage. This force was applied to induce filopodia growth, but in contrast with the results obtained from experiments using fibronectin-coated beads (Supplementary Movie 10 and 11), under these conditions, filopodium did not grow and formation of membrane tethers occurred instead. After switching off the trap at the time point 3:30 the bead immediately returned to filopodium tip as seen in the next frame (3:32) and than randomly moved in the proximity of filopodium tip (3:32-3:54). Thus, the bead remained associated with filopodium even though the link connecting the bead with the filopodium tip became hardly visible because of disappearance of actin. This indicates that the bead and filopodium tip remain connected via the membrane tether. See Methods for details on experiments using optical trap. The duration of the movie is 3 min 54 s and the movie images were recorded at 0.5 fps with a display rate of 15 fps.

Supplementary Movie 13

Inhibition of myosin II suppresses filopodia adhesion and growth (see also Fig. 4b)

Effect of light-insensitive blebbistatin. S-nitro-blebbistatin (20 μM) was added to the cell 20 min prior to placing the laser trapped fibronectin-coated bead onto the filopodium tip. 30 s later, stage movement commenced simultaneously with filming.

Note that adhesion of the bead to filopodium was broken 6 min 44 s after starting the stage movement. The duration of the movie is 13 min 10 s. It was recorded at 0.5 fps with a display rate of 15 fps.

Supplementary Movie 14

Effect of myosin IIA knockdown on filopodia adhesion and growth (see also Fig. 4c)

The detachment of the filopodium from the bead occurred 3 min 50 s after the stage movement was initiated. The velocity of the retrograde movement of the myosin X patch in this experiment was about 10nm/s, significantly lower then the velocity of myosin X patches driven by myosin II (Fig. 5b). The duration of the movie is 4 min 38 s. It was recorded at 0.5 fps with a display rate of 15 fps.

Supplementary Movie 15

Immediate effect of ROCK inhibitor Y-27632 on force-induced filopodia growth and adhesion

This movie corresponds to the frames shown in Supplementary Fig. 2a. HeLa-JW cell filopodium growth was induced by applying a pulling force, generated as a result of microscope stage movement, as in Fig. 3. At about 4 min after stage movement commenced, 50 μM of Y-27632 was added, which eventually resulted in bead detachment at 17 min 25 s. The intensity of actin labeling was relatively low and apparent "disappearance" of actin in the second half of the movie was a result of photobleacning. The duration of the entire movie is 19 min 22 s. The movie was recorded at 1 fps and displayed at 25 fps.

Supplementary Movie 16

Inhibition of formin suppresses filopodia adhesion and growth (see also Fig. 4d)

Effect of formin inhibition by SMIFH2. The cell was pretreated with 40μM SMIFH2 for one hour prior the bead being placed onto the filopodium tip. The detachment of the filopodium from the bead occurred about 3 min 20 s after the stage movement was initiated. The duration of the entire movie is 4 min 56 s. All the movies in this figure were recorded at 0.5 fps with a display rate of 15 fps.

Supplementary Movie 17

Effect of myosin II inhibition on fast centripetal movement of myosin X patches in filopodia of SMIFH2 treated cells (part 1)

This movie corresponds to Fig. 5a, top. A HeLa-JW cell transfected with GFP-myosin X and F-Tractin-tdTomato was filmed before addition of SMIFH2. The duration was 6 min 41 s; the movie images were recorded at 0.1 fps and displayed at 7 fps.

Supplementary Movie 18

Effect of myosin II inhibition on fast centripetal movement of myosin X patches in filopodia of SMIFH2 treated cells (part 2)

These movies correspond to Fig. 5a, middle. The same HeLa-JW cell as in Supplementary Movie 17, 15 min after the addition of 20 μM SMIFH2 was filmed. Note that after SMIFH2 was added, myosin X comet tails underwent rapid disintegration into small patches, which moved centripetally towards the cell body. The duration of the movie was 6 min 41 s; the movie images were recorded at 0.1 fps and displayed at 7 fps.

Supplementary Movie 19

Visualization of photoactivated actin in filopodia expressing myosin X

The left panel corresponds to mApple-myosin X. The right panel represents photoactivated PAGFP-b-actin in the same filopodium of HeLa-JW cell. The site of photoactivation is indicated by the pink line and was performed at 6 s from the start of the movie. Note the retrograde movements of myosin X and actin inside the filopodium. The kymograph of the line drawn along the length of the filopodium, in which the photoactivation assay was performed, is shown in Supplementary Fig. 4b. The duration of the movie is 57 s and the movie images were recorded at 0.6 fps with a display rate of 7 fps.

Supplementary Movie 20

Centripetal co-movement of VASP and myosin X patches in cells treated with formin inhibitor

Co-localization of mApple-myosin X (green) and GFP-VASP (red) in the same filopodia of HeLa-JW cell 90 min after the addition of 20 μM SMIFH2. The duration of the movie is 83 min 10 s and the movie images were recorded at 0.1 fps with a display rate of 50 fps. Movie was obtained by SDCM.

Supplementary Movie 21

Effect of myosin II inhibition on fast centripetal movement of myosin X patches in filopodia of SMIFH2 treated cells (part 3)

These movies corresponds to Fig. 5a, bottom. The same HeLa-JW cell, as in Supplementary Movies 17 and 18, was filmed 20 min after the subsequent addition of 30 μM Y-27632. Note, that the addition of Y-27632 resulted in cessation SMIFH2-

induced centripetal movement of myosin X-positive patches. The duration of the movie was 6 min 41 s; the movie images were recorded at 0.1 fps and displayed at 7 fps.

Supplementary Movie 22

Immediate effect of formin inhibitor SMIFH2 on force-induced filopodia growth and adhesion

40 μM of SMIFH2 was added to HeLa-JW cell expressing GFP-myosin X (green) and F-Tractin-tdTomato (red) after establishment of sustained filopodia growth (at 11th min). About 3 minutes later, cessation of growth of the actin core and the drop in the pulling force generated by the filopodium were observed but the fibronectin-coated bead remained associated with the filopodium tip via a membrane tether. The duration of the entire movie is 20 min 39 s. The movie was recorded at 1 fps and displayed at 30 fps. (A sequence from this movie is shown also in Supplementary Fig. 2c).

Supplementary Movie 23

Formin inhibitor SMIFH2 enhances the detachment of formins from actin filaments *in vitro* (control)

The constitutively active mDia1 formin construct (FH1FH2DAD) was anchored to the glass surface of a microfluidic chamber by one of their FH2 domains using an anti-His antibody. Actin filaments were grown in the presence of Alexa488-labelled actin to form fluorescent segments at the tips of actin filaments with subsequent exposure, from time zero onward, to 1 μM unlabeled actin and 4 μM profiling. The

duration of the entire movie is 4 min 40 s. The movie was recorded at 0.05 fps and displayed at 7 fps. (Frames from this movie are also shown in Fig. 5c, top row).

Supplementary Movie 24

Formin inhibitor SMIFH2 enhances the detachment of formins from actin filaments *in vitro* (treatment)

This movie records the experiment analogous to control (Supplementary Movie 23) conducted in presence of 100 μM SMIFH2 inhibitor in solution. The duration of the entire movie is 6 min 20 s. The movie was recorded at 0.05 fps and displayed at 7 fps. (Frames from this movie are also shown in Fig. 5c, bottom row).

Supplementary Movie 25

Filopodia distinguish between fluid and rigid substrates

GFP-myosin X transfected HeLa-JW cell spreading on the micropatterned substrate with 3 μm islands covered with supported lipid bilayer (SLB). Both the islands and the rigid substrate between them were coated with fluorescent Dylight-405 RGD ligand shown in blue. The GFP-myosin X positive filopodia tips are shown in green. The movie started 30 min following the cell plating. Note that filopodia tips apparently avoid the SLB islands, see the analysis in Fig.8. The duration of the movies was 16 min 36 s; the movie images were recorded at 0.3 fps and displayed at 30 fps.

Supplementary Movie 26

Pulling of Cdc42 Q61L-induced filopodia

Filopodia of a Hela-JW cell transfected with constitutively active Cdc42 mutant, GFP-Cdc42 Q61L, and F-Tractin-tdTomato were pulled using the optical trap. Arrow indicates the center of the trap. Note, that Cdc42-induced filopodium attached to laser-trapped fibronectin-coated bead did not grow upon stage movement and eventually pulled the bead out of the trap. The duration of the movie is 6 min 36 s and the movie images were recorded at 1 fps with a display rate of 15 fps.

Supplementary Movie 27

Pulling of mDia2Δ DAD-induced filopodia

Filopodia of a Hela-JW cell transfected with constitutively active mDia2 mutant, mDia2 ΔDAD-GFP, and F-Tractin-tdTomato were pulled using the optical trap. Note, that mDia2-induced filopodium attached to laser-trapped fibronectin-coated bead after around 4 min 30 s from the beginning of the movie started to grow independently of pulling and continued to grow further bypassing the bead location. The duration of the movie is 9 min 30 s and the movie images were recorded at 1 fps with a display rate of 15 fps.
